# Supplementary material for: Supplementation with Octacosanol Affects the Level of PCSK9 and Restore Its Physiologic Relation with LDL-C in Patients on Chronic Statin Therapy
Source: Nutrients. 2021 Mar 10;13(3):903. doi: 10.3390/nu13030903 (PMC8001635; doi:10.3390/nu13030903)
Supplement: Supplementary file 1 [file nutrients-13-00903-s001.zip › Suppl/Table S1.docx]

**Supplementary Table 1.** Serum aminotransferases and CK levels at baseline, after 8 weeks and 13 weeks of supplementation

| Variable | Placebo (*n* = 44) | | | Dietary supplement (*n* = 37) | | | ANOVA (*p*) | | |
| --- | --- | --- | --- | --- | --- | --- | --- | --- | --- |
|  | Baseline | 8 weeks | 13 weeks | Baseline | 8 weeks | 13 weeks | T | S | T x S |
| ALT (U/L) | 22.60 ± 0.94 | 21.91 ± 0.93 | 22.13 ± 0.96 | 21.22 ± 1.02 | 19.24 ± 1.01 | 19.03 ± 1.04^*^ | 0.049 | 0.049 | 0.357 |
| AST(U/L)^†^ | 24.10 (22.59 - 25.70) | 23.55 (21.93 - 25.29) | 21.63 (20.09 - 23.28)^*^ | 23.11 (21.53 - 24.77) | 22.28 (20.56 - 24.10) | 19.68 (18.20 -21.33)^** #^ | 0.000 | 0.641 | 0.120 |
| CK (U/L)^†^ | 99.54 (83.37 -118.85) | 111.94 (93.11 - 134.28) | 105.93 (88.92 - 126.18) | 107.15 (88.10 - 130.02) | 105.68 (86.50 - 128.82) | 98.17 (81.28 - 118.85) | 0.529 | 0.858 | 0.413 |

Values are shown as mean±SEM or † geometric mean values (95th confidence interval), as appropriate. T-time, S-supplementation, T × S-time and supplementation interaction effect; ALT: Alanine aminotransferase; AST: Aspartate aminotransferase; CK: Creatine phosphokinase.

*p < 0.05, **p < 0.01, ***p < 0.001 compared to baseline, # p < 0.05 versus 8 weeks (*post-hoc* pairwise comparisons with Bonferroni correction).
