# Supplementary figures and images for: Supplementation with Octacosanol Affects the Level of PCSK9 and Restore Its Physiologic Relation with LDL-C in Patients on Chronic Statin Therapy
Source: Nutrients. 2021 Mar 10;13(3):903. doi: 10.3390/nu13030903 (PMC8001635; doi:10.3390/nu13030903)

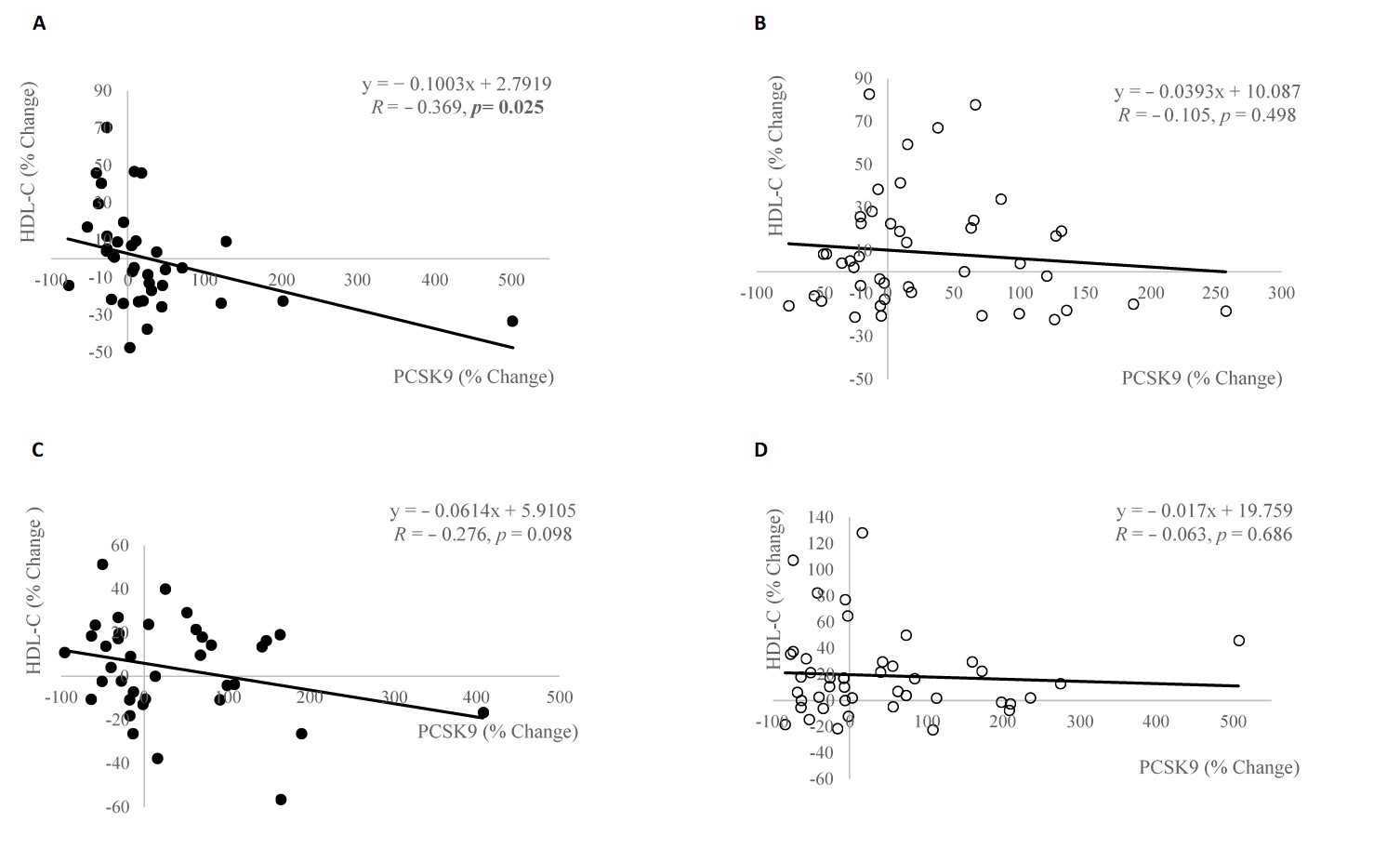

Supplement: Supplementary file 1 [file nutrients-13-00903-s001.zip › Suppl/Figure S1.jpg]
